# Microbiota-derived extracellular vesicles: current knowledge, gaps, and challenges in precision nutrition

**DOI:** 10.3389/fimmu.2025.1514726

**Published:** 2025-02-20

**Authors:** Elvira Marquez-Paradas, Maria Torrecillas-Lopez, Luna Barrera-Chamorro, Jose L. del Rio-Vazquez, Teresa Gonzalez-de la Rosa, Sergio Montserrat-de la Paz

**Affiliations:** ^1^ Department of Medical Biochemistry, Molecular Biology, and Immunology, School of Medicine, University of Seville, Seville, Spain; ^2^ Instituto de Biomedicina de Sevilla, IBiS/Hospital Universitario Virgen del Rocio/CSIC /Universidad de Sevilla, Seville, Spain

**Keywords:** diet, immunity, immunonutrition, gut microbiota, gut-brain-axis

## Abstract

The gut microbiota has co-evolved with its host, profoundly shaping the development and functioning of the immune system. This co-evolution has led to a dynamic relationship where microbial metabolites and molecular signals influence immune maturation, tolerance, and defense mechanisms, highlighting its essential role in maintaining host health. Recently, bacterial extracellular vesicles (BEVs), membrane nanoparticles produced by bacteria, have emerged as important players in gut balance and as potent immune modulators. These vesicles reflect the characteristics of the bacterial membrane and contain nucleic acids, proteins, lipids, and metabolites. They can regulate immune processes and are involved in neurological and metabolic diseases due to their ability to distribute both locally in the gut and systemically, affecting immune responses at both levels. This review provides a comprehensive overview of the characteristics and functional profile of BEVs, detailing how nutrition influences the production and function of these vesicles, how antibiotics can disrupt or alter their composition, and how these factors collectively impact immunity and disease development. It also highlights the potential of BEVs in the development of precision nutritional strategies through dietary modulation, such as incorporating prebiotic fibers to enhance beneficial BEV production, reducing intake of processed foods that may promote harmful BEVs, and tailoring probiotic interventions to influence specific microbial communities and their vesicular outputs.

## 1 Introduction

The gut microbiota is defined as the set of microorganisms that colonise the gastrointestinal tract of mammals and is estimated to consist of about 100 trillion cells, the majority being bacterial species with a minority of fungal, archaeal, or even viroid kingdoms (1). In recent decades, extensive research has shown that the gut microbiota plays a key role in host physiology and immunity (2), as this complex ecosystem has essential functions in processes such as digestion and metabolism, immune system development and balance, maintenance of gut barrier integrity and functionality, angiogenesis, bone health, and behavior (3–6). Because of all its implications, the detrimental alteration of the composition or functionality of the gut microbiota, known as dysbiosis, is linked to the development or progression of numerous pathological processes such as cancer, inflammatory bowel disease, and metabolic disorders like obesity and diabetes (7–9). Of the many possible endogenous and exogenous host factors involved, diet emerges as a fundamental influencer of the gut microbiota community, which can determine not only the composition or diversity of the gut microbiota, but also the functionality and methodology of microbiota-gut interaction. Understanding the mechanisms by which these microorganisms regulate physiological processes and the role of different dietary signals in this ecosystem is crucial for developing effective therapeutic strategies against many diseases. Among the many research, at the height of the potential of extracellular vesicles (EVs), bacterial extracellular vesicles (BEVs) have emerged as important mediators of microbe-host communication. In this review, we analyze current knowledge on the biogenesis and content of BEVs, their role in host physiology and pathology, and the factors and dietary patterns that may influence their characteristics and functionality. We also address key gaps in understanding, such as the mechanisms governing BEV-host specificity and their systemic effects, as well as challenges in translating these findings into precision nutritional strategies.

## 2 BEVs, an unexplored communication system

The gut microbiome is primarily composed of bacteria. There are estimated to be approximately 1000 bacterial species (10), most of which are present and common to all humans, although a minority is dependent on environmental and genetic factors, making the microbiota a dynamic and heterogeneous organ of high complexity (11). In 1967, electron microscopy revealed the production of extracellular vesicles (EVs) in bacteria, later termed bacterial extracellular vesicles (BEVs). Initially regarded as waste products, this discovery marked a turning point in understanding bacterial communication, paving the way for decades of research that redefined their biological significance (12). Continuing scientific advances have changed the perception of the usefulness of these BEVs. Although the fate and actions of these vesicles in host cells remain unexplored territory, recent decades have been significant progress in elucidating the mechanisms of their biogenesis. Furthermore, the composition and functionality that characterize them have become clearer.

### 2.1 General properties of BEVs

**Figure 1:**
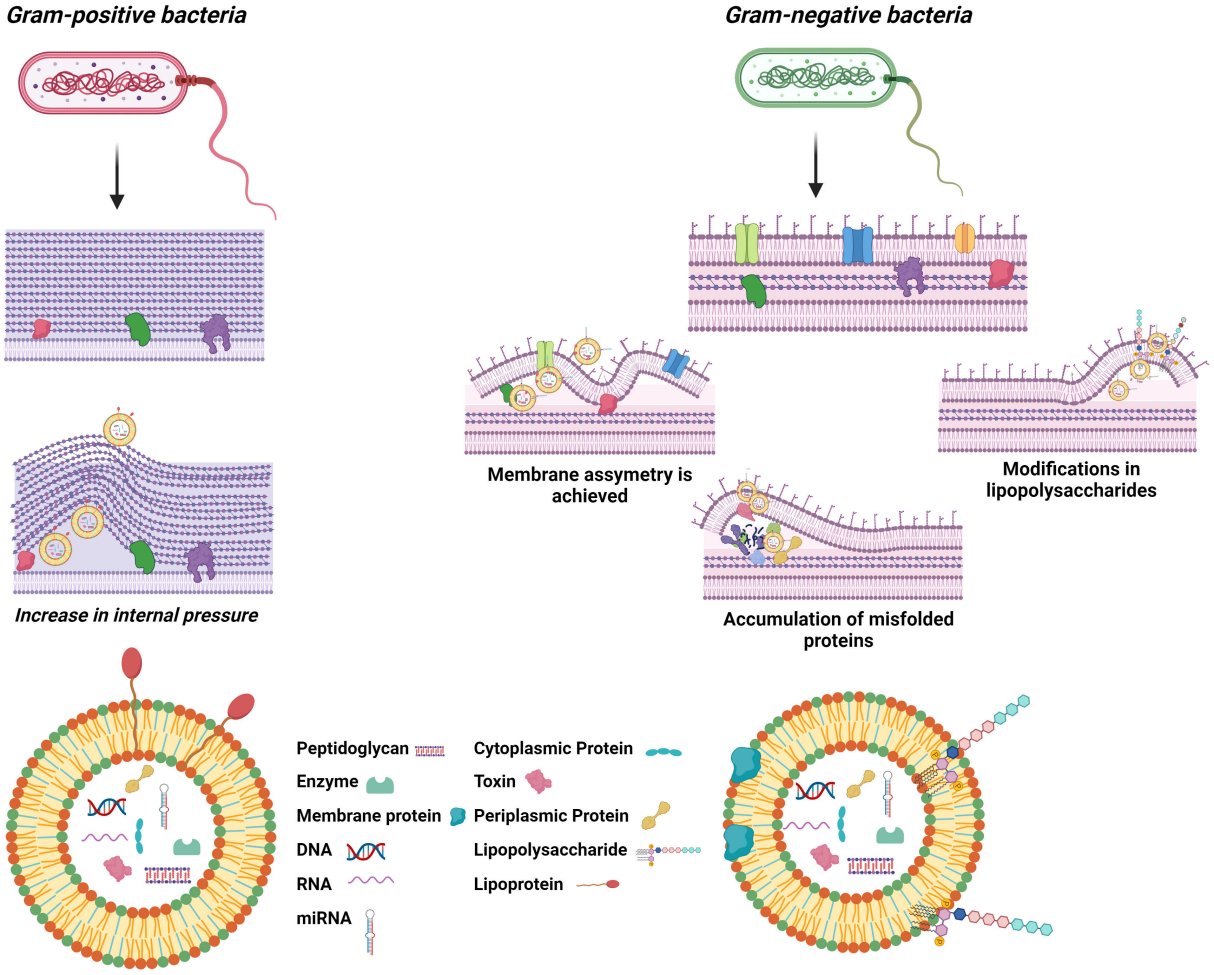
Schematic description of the general structure of BEVs and their biogenesis.

BEVs are nanometer-sized vesicles that are formed from the parent cell membrane of the cell of origin. Unlike eukaryotic EVs, BEVs range in size exclusively below 400 nm in diameter, and the biogenesis process, structure, and content also differ from them, although they all consist of a lipid bilayer that acts as a container for several molecules (13). The biogenesis method depends on the type of bacteria, as their structural differences are crucial for understanding BEV formation. Gram-negative bacteria consist of a double layer of plasma membrane separated by the periplasmic space, a peptidoglycan polymer attached to the inner part of the outer membrane, and a lipopolysaccharide (LPS) anchored to the surface (14). These structural components enable the formation of outer membrane vesicles (OMVs) directly from their outer membrane, a process influenced by membrane stability and stress responses. In contrast, Gram-positive bacteria, with their thick peptidoglycan layer, rely on entirely different mechanisms, such as potential inner membrane rupture, for vesicle formation. Although there are discrepancies, three hypotheses appear to have been suggested that could explain OMV production; OMVs are produced when the lipid asymmetry of the outer membrane is compromised, when there is accumulation of misfolded proteins in the outer membrane, or when LPS modifications occur, and all seem to respond to a mechanism of outer membrane homeostasis maintenance (**Figure 1**) (15). The first hypothesis defends that the stability of the bacterial envelope is maintained by organised interactions between the lipids of the outer membrane, peptidoglycan, and some associated proteins such as OmpA or Braun lipoprotein (16). When certain factors alter these stable interactions, vesicle formation may increase, as has been shown in some bacteria such as E. coli (17). The second hypothesis, on the contrary, argues that the inability to properly handle extracytoplasmic stress responses through pathways such as σE and Cpx (18), promotes vesicle release by allowing the accumulation of misfolded proteins (19). Finally, the last hypothesis explains that bacterial membrane stability depends on interactions between LPS molecules based on salt bridges formed by cations such as Mg²^+^ and Ca²^+^. Thus, any factor affecting LPS-LPS interactions, thus structure or composition, could lead to increased vesicle release (20). On the other hand, gram-positive bacteria have only a thick, stiff outer peptidoglycan layer which was initially thought to impede the release of EV (21). The discovery of the existence of these vesicles is more recent, and they are called bacterial membrane vesicles (BMVs). The mechanism of production is not yet well defined, but it seems that BMVs could be formed by forcing the inner membrane to rupture under increased pressure inside the cell (**Figure 1**) (22). In either case, the synthesis and release of these vesicles appears to be constitutive, although influenced by environmental stress factors or conditions, and although the molecular mechanisms of release of these vesicles to the exterior are not yet fully understood, it is known that they all nanoencapsulate molecules such as nucleic acids, proteins, lipids, and metabolites capable of exerting a function in the target cell during their formation (23). The main differences between vesicles derived from eukaryotic cells and vesicles derived from prokaryotic cells are summarized in **Table 1** (24, 25).

**Table 1:** Summary of the main differences between vesicles derived from eukaryotic cells and vesicles derived from prokaryotic cells.

		*EVs*	*BEVs*
*SIMILARITIES*	*Heterogeneity*	Highly heterogeneous composition of the surface and the interior of the gallbladder	
	*Cargo*	EVs can contain RNA, such as miRNA or mRNA, DNA, proteins and metabolites	
	*Spontaneity*	Non-spontaneous biological process	
	*Release*	Not homogeneously released through the membrane	They are not released evenly through the membrane, there are hot spots
	*Main function*	Intercellular communication	
*DIFFERENCES*	*Size*	Particles range from 500-2000 nm (apoptotic bodies), 100 and 1000 nm (MVs) and 30-100 nm (exosomes).	Particles range from 10 to 400 nm
	*Cell of origin*	Exosomes and MVs can be released from healthy or damaged cells. Apoptotic bodies are released from dying cells	BEVs are released by both gram-negative and gram-positive bacteria
	*Formation*	Apoptotic bodies and MVs originate from the plasma membrane, and exosomes via the endocytic pathway	Gram-negative and Gram-positive bacteria have a different mechanism of formation due to their different membrane structures
	*Markers*	There are universal markers such as CD40 for MVs or flotillin for exosomes	There are no universal markers due to the high diversity of markers
	*Vesicles production*	Level of production depends on the origin cell and the physiology state	Production increases as a response to environmental stress

### 2.2 Composition of BEVs

The effects of BEVs upon release will depend on their cargo, and the composition of the cargo is influenced by the conditions of the producing cells, by environmental or growth conditions, and by external factors. It has been shown that, like eukaryotic EVs, BEVs are rich in proteins. Although their content depends on the taxonomic group of the progenitor bacteria or their specific characteristics, proteomic analyses have already associated them with more than 3000 proteins. These include structural proteins, porins, ion channels or transporters, enzymes, and proteins related to the response to the environment. For example, gram-negative bacteria contain a high concentration of membrane proteins such as OmpA, OmpC, and OmpF, and periplasmic proteins such as AcrA and alkaline phosphatase (26). Proteins involved in the biogenesis or phenotype of the OMVs they release have also been identified, such as rfaE and waaC, involved in LPS synthesis, or mrcB, involved in peptidoglycan synthesis and remodeling (27). These proteins are critical because they regulate the structural integrity and functionality of the vesicles, influencing their ability to interact with host cells and contribute to bacterial survival and pathogenicity. Other recent analyses have also confirmed the presence of proteins involved in colonization, competition, bacterial survival, and regulation of immune processes that take place in the intestinal lumen. Although the protein content of gram-positive BEVs has been less studied, membrane and cytoplasmic proteins involved in numerous biological processes have also been identified. Furthermore, as with BEVs released by gram-negative, the protein content is also influenced by the pathogenicity of the strain. Pathogenic strains often produce BEVs enriched with virulence factors and toxins, which can modulate the host immune system, disrupt cellular processes, and contribute to disease development by facilitating bacterial invasion or evading immune defenses (28, 29).

Lipids also play an essential role, as they are the most important structural component, and are derived from the cytoplasmic membrane and endomembrane. Lipid composition appears to be conserved, including LPS, phosphatidylethanolamine, phosphatidylglycerol, and cardiolipin (30). However, some lipids may be selectively enriched in BEVs, such as phosphatidylglycerol and stearic acid, which are involved in membrane fluidity and rigidity (31). Therefore, lipid species and their distribution seem to depend on the bacterial species, but many of them seem to be related to the adaptation and survival of bacteria in their usual niches (12). For example, phosphatidylglycerol contributes to membrane stability in harsh conditions, while cardiolipin plays a role in energy metabolism and membrane curvature during cell division, aiding bacterial survival.

BEVs also allow transport of nucleic acids to their specific intracellular receptors in the host cell. The presence of both chromosomal and plasmid DNA has been confirmed, which may be involved in immunoregulation, biofilm formation, adhesion, and pathogenicity (32). Numerous studies have formed that BEVs contain DNA in the lumen and on the surface that they transport to other bacteria by horizontal transfer and that corresponds to genes involved in antibiotic resistance, stress, and virulence (33). Recent evidence has shown that BEV-associated mRNAs can be translated to produce microbial proteins in target cells. As in eukaryotic EVs, the content of greatest interest involves miRNAs, which participate in the regulation of post-transcriptional gene expression, inducing changes in the phenotype and immune response of the target cell (34). In addition, these small molecules appear to have the capacity to regulate gene expression in regions involved in epigenetic control mechanisms (35). Although more and more are known about the cargo carried by BEVs, it is essential to characterize these vesicles taking into account the bacterial species of origin and the microenvironmental conditions that may alter the content to provoke one response or another in the host cell (36).

### 2.3 BEV interaction with host-cell

Once BEVs are released outside the cell, they can exert their effect on the target cell by two main mechanisms: direct interaction with the membrane, or by delivery of the bioactive cargo from the membrane (37). Direct interaction with the membrane involves binding of BEV surface molecules to specific receptors on the target cell, leading to signal transduction and activation of downstream pathways. In contrast, delivery of bioactive cargo allows internalization of vesicular contents, such as proteins, nucleic acids, or lipids, which can directly alter cellular processes within the target cell. Direct membrane interaction takes place because the membrane of BEVs contains the same molecules as the plasma membrane of the cell of origin; LPS in the case of gram-negative bacteria and lipoteichoic acid in the case of gram-positive bacteria, capable of activating TLR4 and TLR2 receptors, respectively (12). Although these are the molecules commonly involved in binding, it is known that BEVs can also interact with nucleotide-binding oligomerization domain proteins in the target cell, among others (38). The capacity with which the vesicles interact with these receptors varies depending on the species of origin. This difference influences not only the binding affinity but also the mechanism of endocytosis or uptake of the vesicles by eukaryotic cells. In addition to acting on specific receptors, BEVs can also internalize their bioactive cargo into eukaryotic, leading to various physiological alterations. For instance, their miRNA content can activate or modulate transcription, influencing gene expression. Additionally, BEVs contain proteases and phosphatases capable of triggering the activation or degradation of host proteins. Furthermore, metabolic enzymes within BEVs can modulate DNA synthesis, thereby affecting cellular replication and repair processes (12). However, although the BEV-eukaryotic cell interaction seems to be the most important in the human physiological balance, intrabacterial interaction through BEVs is also essential. These interactions help maintain integrity by facilitating communication between bacterial species, ensuring proper distribution of resources, and regulating growth dynamics to prevent overgrowth or dominance of certain species. This balance is critical for sustaining the diversity and functionality of the microbial community that constitutes the gut ecosystem, as will be discussed below (39).

## 3 Impact of BEVs on human physiology

Knowing the types of interaction, we can classify the functions of BEVs into two groups: those related to bacteria-bacteria interaction and those related to bacteria-host communication.

### 3.1 Bacteria - bacteria interactions

The main objective of the release of BEVs by commensal bacteria is population maintenance through ecological niche persistence. For example, BEVs have the ability to sequester phages to avoid direct interaction with bacteria (39), thereby protecting bacterial populations from viral attacks. They can also act as decoys for harmful substances or antibiotics travelling towards the membrane (12), reducing the direct impact of these agents on bacterial cells. Additionally, by directly transporting enzymes or transferring genes involved in antibiotic resistance (40), BEVs contribute to the spread of resistance traits, enhancing the adaptability and resilience of bacterial communities, which is crucial for maintaining the stability of the ecosystem. In the same way, these vesicles are rich in Quorum Sensing (41), molecules that coordinate bacterial growth and behavior according to population density and promote biofilm formation (42). BEVs can also promote the proliferation of bacterial kingdoms and colonization of the intestinal niche by being loaded with adhesion factors or confer metabolic advantages by being loaded with molecules that facilitate the bacterial nutrient acquisition process, such as hydrolases that degrade complex proteins and polysaccharides or amino acid or fatty acid transport systems (43). **Table 2** summarizes some of the findings that support the importance of microbe-microbe.

**Table 2:** BEVs in cross-communication between intestinal bacteria.

Origin	Function	Cargo	Evidence
*Pseudomonas areuginosa*	Coordination of bacterial interaction activities, iron acquisition,	2-heptyl-3-hydroxy-4-quinolone	(44)
	Antibiotic resistance	β-lactamases, membrane-bound proteases	(45)
	Lysis of other microbes	Proteases, hydrolases, bacteriocins	(46)
	Formation of biofilms	DNA and PQS	(47)
*Escherichia coli*	Protection of commensal bacteria from harmful substances	LPS and other unknown factors	(48)
	Antibiotic resistance	β-lactamases, membrane-bound proteases	(49)
	Horizontal transfer of virulence genes	DNA for intimin and Shiga toxin encoding genes	(50)
	Horizontal transfer of antimicrobial resistance genes	DNA for β-lactamase genes	(50)
*Bacteroides Fragilis*	Lipopolysaccharide degradation	Acid lipoproteins	(51)
*Vibrio cholerae*	Protection of commensal bacteria from harmful substances	PrtV protease	(52)
	Inhibition of innate immune response	Unknown	(53)
*Bacteroidetes thetaiotaomicron*	Protection of commensal bacteria from antibiotics	β-lactamase	(40)
	Degradation of polysaccharides	Lipoproteins	(51)
*Bifidobacteria longum*	Promotion of Bifidobacteria colonisation	Mucin-binding proteins	(54)
*Helicobacter pylori*	Protection against oxidative damage	KatA	(55)
	Biofilm formation	DNA and others	(56)

### 3.2 Bacteria – host interactions

On the other hand, BEVs can interact with intestinal eukaryotic cells. Although the most studied actions have been on the epithelial cells lining the intestinal barrier, some studies have shown that these vesicles cross the mucosal barrier to modulate the direct action of cells of the innate and adaptive immune system. Although the regulatory effects of BEVs depend on the bacteria of origin, host physiology and the cargo they contain, they are known to play a crucial role in the maintenance of intestinal immune homeostasis. The usual mechanism of action is to trigger an immune response in the host cell due to the fact that they contain molecular patterns (PRRs), such as proteins, DNA, or RNA, capable of interacting with pattern recognition receptors such as NOD1 and NOD2 and TLRs, initiating signaling cascades that regulate immune responses (57). These interactions will result in the triggering of signaling pathways, synthesis of pro-inflammatory or anti-inflammatory cytokines, polarization of macrophages to anti-inflammatory phenotypes, differentiation of B cells into plasma cells, promotion of the regulatory immune response, etc (58). Some examples of BEV action can be found in **Table 3**. In this sense, when BEVs are released by commensal bacteria, the main objective is to modulate and balance innate and adaptive immunity to promote host defense against pathogenic bacteria and maintain intestinal microenvironmental stability by regulating metabolic and energetic processes. However, the effects of BEVs can be turned against us when they are released by pathogenic bacteria or when dysbiosis allows uncontrolled passage into systemic circulation, which is why BEVs are being studied as key players in the development and progression of numerous pathologies.

**Table 3:** BEVs in communication cross-talk between gut bacteria and host cells.

Origin	Function	Mechanism	Evidence
*Bacteroidetes fragilis*	Immunomodulation	Induction of Treg and IL-2 production through TLR2	(59)
	Complex carbohydrate metabolism	Packaging and surface exposure of lipoproteins	(43)
*Akkermansia muciniphila*	Attenuating the progression of induced colitis	Decreased IL-6 production	(60)
	Improving the integrity of the intestinal barrier	Increased binding proteins (occludin, ZO-1 and claudin-5)	(61)
	Increasing levels of 5-HT in the intestinal lumen	Modulation of genes involved in serotonin synthesis	(62)
	Improving obesity and associated complications	Reprogramming of pro-inflammatory cytokines and modulation of genes involved in energy metabolism (PPAR-α and PPAR-γ).	(63)
*Escherichia coli*	Improvement of intestinal barrier integrity	Increased expression of ZO-1 and claudin-14	(64)
	Immunoregulation	Activation of NF-kB and secretion of proinflammatory cytokines IL-6 and IL-8	(65)
	Attenuation of induced colitis progression	Positive up-regulation of IL-10; down-regulation of IL-1β, TNF-α, IL-6, IL-12, IL-17, iNOS and COX-2 in colonic tissue	(66)
*Pseudomonas panacis*	Blockade of insulin uptake by myotubes and induction of diabetic phenotype	Downregulation of the insulin signaling molecule pAKT and GLUT4 translocation	(67)
*Pseudomonas Aeruginosa*	Activation of apoptosis and inflammation	Induction of macrophage dysfunction and inhibition of protein synthesis	(68)
*Pediococcus pentosaceus*	Disturbance of skin healing processes	Inflammatory suppressor cell recruitment and differentiation	(69)
	Inhibition of inflammation	Induction of macrophage polarization to M2	(69)
*Bacteroidetes thetaiotaomicron*	Development of colitis	Induction of the production of inflammatory cytokines by intestinal macrophages	(70)
	Regulation of energy metabolism	Induction of cholesterol uptake by up-regulation of NPC1L1	(71)

## 4 BEVs, from friend to foe

Disruption of proper communication between species in the gut microbial niche can be the cause or consequence of numerous pathologies. Given that the intestinal barrier acts by forming a physical and biochemical defense that prevents the translocation of microbes, toxins, and antigens from the gut into other organs and tissues, most research has focused on understanding disorders and diseases of intestinal origin, such as inflammatory bowel disease (IBD), colitis, Crohn’s disease, and colorectal cancer (72). As mentioned above, BEVs are known to play a key role in the maintenance of intestinal integrity. However, they are also recognized as inflammatory factors in the context of the dysbiotic microenvironments that characterize intestinal pathologies. For example, in the context of the characteristic microbial alterations that define IBD, an increase in gram-negative bacteria is often observed. This microbial imbalance leads to an abundance of LPS-rich BEVs has been observed that can penetrate epithelial cells, triggering inflammatory responses and altering the expression of genes involved in barrier maintenance (73). In addition, BEVs derived from a dysfunctional microbiota may promote IBD progression by increasing the release of EVs from the intestinal mucosa with high concentrations of inflammatory agents such as CCL20 and prostaglandins E2 (74). Recently, BEVs from bacteria such as *Bacteroides fragilis* have also been shown to contain toxins capable of breaking down E-cadherin and triggering the release of IL-8. This would mainly affect patients with colorectal cancer, as they generally have an increase of bacteroidetes in their microbiota that promote chronic inflammation and disruption of the intestinal barrier (74). However, we now know that the capacity of these vesicles goes far beyond this, and they are now linked to numerous systemic diseases. In this section we will discuss the current knowledge on BEVs in metabolic disease and neurological disease.

### 4.1 Metabolic syndrome

Metabolic syndrome (MetS) and its associated complications, such as obesity, currently affect 25% of the world’s population and are the first step in the development of cardiovascular disease (75). Research and advances in this field have grown enormously in recent years, particularly in understanding the microbiota’s role in metabolic dysregulation. These advances include identifying how microbial diversity impacts energy balance, immune regulation, and the systemic effects of microbiota-derived metabolites and vesicles. The findings of the close relationship between these disorders and the disruption of the gut microbiota balance have prompted the development of numerous metagenomic studies in recent years (76). The main notable change affects the two most abundant phyla of the microbiota: Bacteroidetes (gram-negative) and Firmicutes (gram-positive). Under normal conditions, the correct proportions of these two phyla maintain intestinal health by properly regulating energy metabolism and maintaining the balance of the immune system. However, in people with these metabolic disorders, a decrease in the abundance of Bacteroidetes and an increase in the abundance of Firmicutes has been observed, which is directly associated with metabolic endotoxemia (77). Metabolic endotoxemia is the consequence of increased permeability caused by dysregulation of microbiota, and is characterized by a passage of PAMPs, metabolites, and BEVs into the systemic circulation, mainly leading to the chronic low-grade immune activation observed in obese subjects (78). For example, translocation of PAMPs contained in BEVs such as LPS interact with TLR4, triggering signaling pathways aimed at NF-кB activation, and the production of cytokines and chemokines. This process promotes macrophage polarization towards an inflammatory phenotype, which subsequently alters the normal functioning of organs such as liver, skeletal muscle, and adipose tissue (79). Indeed, BEVs produced by *Pseudomonas aeruginosa*, whose concentration in LPS and other protein components are able to stimulate the production of TNFα, IL-6, IL-1β, and stimulate macrophage polarization (80). One of the most studied examples in relation to metabolic disorders is that of BEVs derived from *Akkermansia muciniphila*. BEVs derived from this species have recently been shown to have beneficial effects on the integrity of the intestinal barrier, as treatment of CACO-2 with these vesicles has been shown to decrease its permeability by stimulating occludin synthesis and treatment of colonic epithelial cells decreases IL-6 production (60). Metagenomic data have demonstrated the inverse correlation between *A. muciniphila* and disorders such as obesity and diabetes, so many therapeutic approaches aimed at restoring their correct proportions are being proposed. For example, Chelakkot et al. demonstrated that oral administration of *A. muciniphila*-derived BEVs improved intestinal barrier integrity, reduced body weight, and improved glucose tolerance in mice fed with High-Fat Diet (HFD), resulting in improved metabolic functions (61). On the other hand, Choi et al. revealed that *Pseudomonas Panacis*-derived BEVs containing LPS were more abundant in HFD-fed mice (67). They also observed that under this diet, more drastic changes were observed in the BEV profile than in the microbial community composition, and this was associated with altered glucose metabolism by promoting impaired insulin signaling in skeletal muscle and adipose tissue. Taken together, these and other studies highlight the role of BEVs as key contributors to low-grade systemic inflammation in subjects with MetS. BEVs drive this inflammation through mechanisms such as triggering TLR-mediated cytokine production, altering immune cell polarization, and disrupting metabolic pathways in organs like the liver and adipose tissue. These processes underscore their central role in the progression of complications like diabetes and obesity. Therefore, focusing on characterizing the profile of these vesicles will allow us to direct attention to the design of treatments aimed at restoring the balance of this ecosystem, as the plasticity of the microbiota to modulable factors such as diet or exercise may open up a field of interest for the design of nutritional interventions aimed at improving or fully restoring the microbial composition and the functionality and content of these BEVs.

### 4.2 Neurological diseases

The relationship between the microbiota-gut-brain axis and its relation to neurological diseases and behavioral disorders has been increasingly studied over the last decade, with a focus on its roles in regulating neurotransmitter production, immune modulation, and endocrine signaling. The term refers to the set of interactions established between the gut microbiota and the central nervous system (CNS) through immunological mechanisms and endocrine signaling, and although hundreds of studies now support the existence and importance of this axis, the mechanisms of signal transfer have yet to be fully elucidated (81). One of the pathways of communication between the two organs is through BEVs, and there is some evidence to confirm that this may occur via four possible mechanisms (**Table 4**). The first mechanism involves the stimulation of the vagus nerve (86), which facilitates communication between the enteric nervous system and the gut microbial community. The second is the endocrine response, wherein bacteria to influence the production of hormones and other chemical signals that travel through the circulation (82). The third mechanism is the inflammatory response triggered at the systemic level (84). Finally, the fourth involves the direct delivery of cargo from these vesicles to the CNS after travelling through the systemic circulation (83). Taken together, the role of BEVs as direct cargo transporters to the brain seems to be the most studied. BEVs have been shown to have the ability to cross the blood-brain barrier and can harbor numerous psychoactive molecules and a wide diversity of neurotransmitters, such as dopamine, noradrenaline, serotonin, and enzymes involved in their synthesis (85, 87). For example, Zakharzhevskaya et al. showed that strains of *Bacteroides fragilis* produced BEVs rich in histamine and GABA. Histamine, in addition to having local functions in regulating gut and immune function, is capable of influencing brain functions related to sleep, learning and anxiety, among others (88). GABA, on the other hand, is the main inhibitory neurotransmitter, and its altered levels have been linked to depressive disorders, so some groups point to the use of these vesicles as a therapeutic target in neurological disorders (89). But neurotransmitters are not the only cargo of BEVs, as they are rich in RNA molecules and proteins that may exert key functions. RNAs play an essential role in acting as regulators from host target genes and as potential epigenetic modulators, which are directly related to synaptic plasticity (35). Following this line, Emery et al. assessed that in postmortem brains of Alzheimer’s subjects, vesicles contained a higher amount of actinobacteria and firmicutes-derived RNA than healthy controls, as well as a depletion of RNA from proteobacteria and *Bacteroidetes* (83). *Aggregatibacter actinomycetemcomitans*-derived BEVs also contain RNAs that appear to act in the pathogenesis of Alzheimer’s disease by stimulating Toll 8 and NF-кB signaling pathways (90). On the other hand, Zhan et al. also observed that *E. coli* K99-derived LPS appeared in the grey matter of Alzheimer’s subjects compared to healthy controls. As for BEV signaling through the enteric nervous system, evidence is limited (91). A recent study suggested that BEVs derived from the intestinal bacterium *Paenalcaligenes hominis* might stimulate cognitive impairment via the vagus nerve, causing cognitive deficits and activation of microglia in the hippocampus (86). Khalid Al-Nedawi et al. found that BEVs derived from *L. rhamnosus* increased the excitability of neurons in the myenteric plexus to send signals through the vagus nerve and alter brain activity and behavior (84). On the other hand, the existence of endocrine signaling is demonstrated by studies such as that of Choi et al., who identified that BEVs from *Lactobacillus plantarum* positively regulated brain-derived neurotrophic factor transcription in hippocampal cells and improved stress-induced behaviors in restrained mice (92). Likewise, Yaghoubfar et al. found that BEVs derived from *A. muciniphila* increased serotonin levels in the colon and hippocampus of mice, thus affecting endocrine serotonin signaling through the gut-brain axis, which may contribute to the pathogenesis of various disorders (82). Commensal bacteria such as *A.* muciniphila or *Bacteroides Fragilis* have the ability to release BEVs rich in molecules to modulate the inflammatory response at the systemic level, inducing the activation of anti-inflammatory cytokines and inhibiting the production of pro-inflammatory cytokines under physiological conditions. Conversely, certain pathogenic bacteria have the capacity to release LPS-rich BEVs and other PRRs capable of activating the immune system at the systemic level. Immune activation has been linked on numerous occasions to learning, anxiety and memory disorders (89, 93). This suggests that BEV interactions with the immune system contribute to the ability of gut bacteria to modify host brain function and behavior.

**Table 4:** Summary of the main signaling mechanisms of BEVs across the gut-brain-axis.

Signaling mechanism	Description	Evidence
Vagus nerve stimulation	BEVs and metabolites produced by intestinal bacteria can cause changes in the activity of the enteric nervous system, which stimulate the vagus nerve modulating the signals reaching the brain and consequently, its activities and functions.	(82)
Modulation of immune signals	BEVs and other metabolites can cause systemic immune activation, leading to the synthesis of molecules such as cytokines capable of crossing the blood-brain barrier to affect neuronal function and neuroinflammation.	(83)
Modulation of endocrine signals	BEVs and bacterial metabolites influence the production of hormones and other chemical signals that are able to travel through the bloodstream to the brain and modulate CNS activity.	(84)
Cargo transport	BEVs released by the microbiota act as transporters of molecules able to travel through the systemic circulation to interact directly with the CNS.	(85)

Taken together, this supports the hypothesis that BEVs are signaling molecules capable of controlling brain activities and normal neurological functioning, being a key component in the development and progression of neurological and behavioral diseases. Further omics studies are required to elucidate the provenance of BEVs found at the systemic level and the bioactive cargo capable of altering physiological molecular mechanisms. Techniques such as transcriptomics can provide insights into RNA profiles associated with BEVs, while proteomics can identify functional proteins involved in signaling pathways. Metabolomics may also uncover bioactive metabolites carried by BEVs, shedding light on their systemic effects. In addition, a better understanding of the biological mechanisms controlling the synthesis and packaging of molecules in these vesicles opens the possibility of exploiting BEVs as drug delivery platforms for specific targets.

## 5 Diet, the perfect ally to tame our gut microbiota

**Figure 2:**
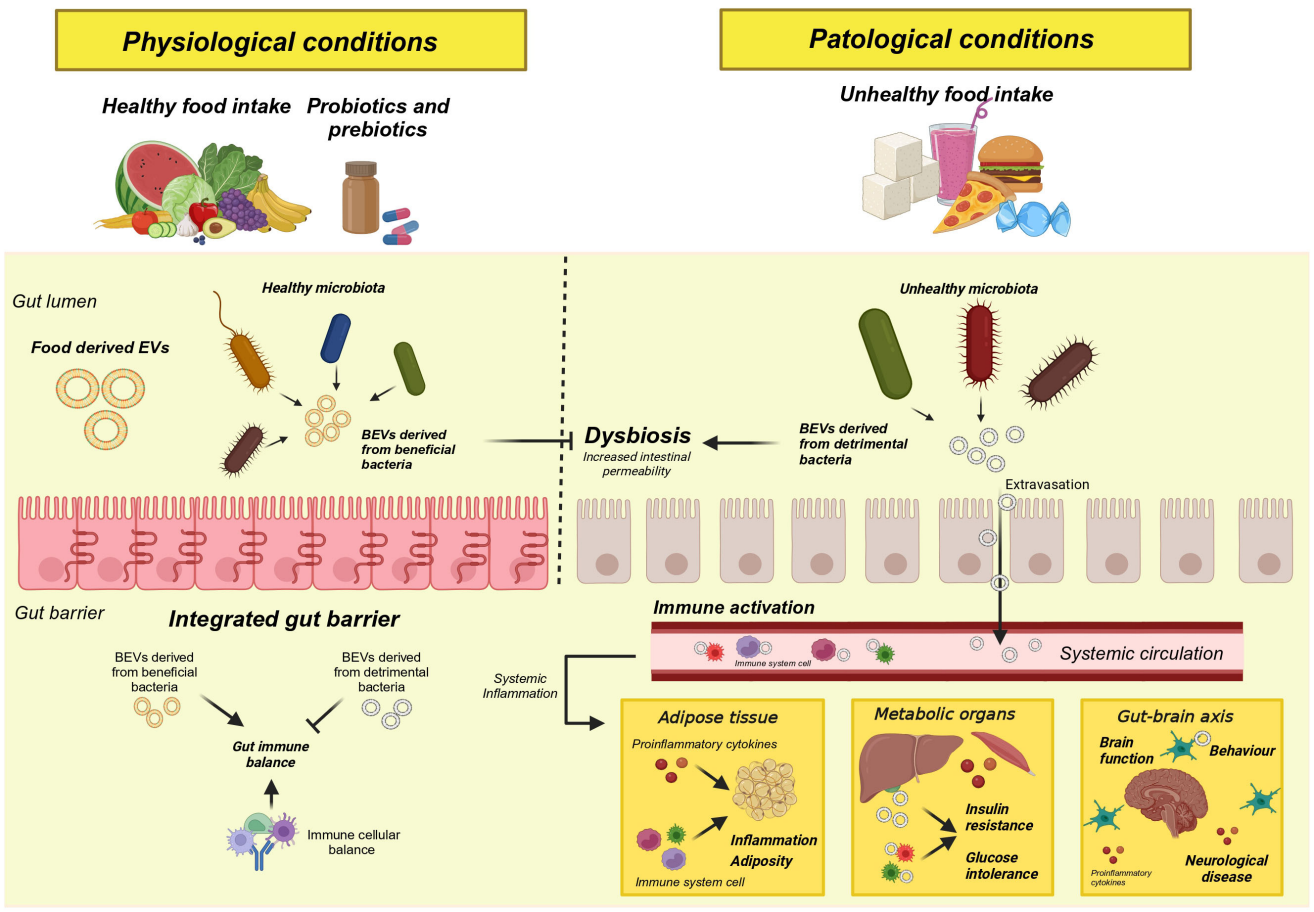
Graphical representation of the consequences of a disruption of the gut microbiota.

If there is one advantage to disrupting the normality of the gut microbiota, it is that it is easily modulated through environmental factors such as diet, exercise, and pre/pro/postbiotics. In recent years, compelling evidence has been found to demonstrate the essential role of the food we eat in the composition, functionality, diversity and abundance of species in the microbiota. For instance, studies have shown that high-fiber diets promote the growth of beneficial bacteria like Bifidobacterium and Lactobacillus, while Western diets rich in saturated fats and refined sugars lead to a decline in microbial diversity and an increase in pro-inflammatory species such as Enterobacteriaceae (**Figure 2**). For example, among all the detriments of Western diets, a notable alteration of the intestinal microbiota has been found with a reduction of beneficial and protective species and an increase in their pro-inflammatory functions (94). In contrast, vegetarian diets have the ability to increase the abundance of beneficial bacteria such as *Bacteroides*, *Prevotella*, and *Clostridium*, among others, reducing harmful bacteria with a pro-inflammatory role such as *Enterobacteriaceae* (78). On the other hand, the Mediterranean diet, recognized as one of the healthiest diets in the world, has also demonstrated its beneficial effects by increasing the abundance of beneficial fiber and carbohydrate-degrading bacteria linked to the metabolism of short-chain fatty acids such as butyrate (95). In addition, the identification of molecular content and beneficial properties of vesicles derived directly from food is also a pioneering field of study in the development of new functional food strategies (96). These evidence led us to believe not only that the profile of BEVs derived from the gut microbiota may be altered or modulated by dietary patterns, but also that these BEVs will have a physiological impact on the host depending on the effect or stimulation caused by these same foods. In this section, we will summarize current findings on the influence of different macronutrients on the content and functionality of BEVs.

### 5.1 Proteins

Dietary protein intake can modulate BEV production through succinate production. Specifically, Tan et al. demonstrated that a high-protein diet enhanced BEV production under succinate stress by activating the TLR4 signaling pathway and consequently increasing IgA-producing mechanisms, an antibody essential for intestinal balance by preventing the distribution of pathogens and affecting their viability (97). These results were supported by Luck et al, who observed that a HFD decreased IgA production, altered glucose homeostasis, gut and adipose tissue inflammation, and altered intestinal permeability and pathogen invasion (98). These findings have led to the proposal that the increase in succinate seen in high-protein diets may be beneficial in attenuating metabolic disorders. Isolated amino acids also seem to have their own effect on BEVs. This is the case of glycine, which is able to increase the release of BEVs by *E coli*, with a larger size and an altered protein profile towards an increase in cytoplasmic and inner membrane proteins (99). However, the effects of glycine go further and affect other bacterial species. For example, van de Waterbeemd et al. demonstrated that cysteine deprivation caused growth depletion and release of BEVs by *Neisseria meningitidis*, altering the assembly of iron and sulphur proteins and increasing oxidative stress (100). The same occurred with *Francisella tularensis*, a pathogenic bacterium able to regulate its vesiculation upon glycine deprivation (101). Although much remains to be clarified, the possibility has been raised that even some isomers present in certain amino acids may have an important role in vesiculation through the regulation of peptidoglycan synthesis and structure, so determining the effect of amino acids on the release and functionality of BEVs is essential to better understand the influence and contribution of high-protein diets on the health of the gut microbiota (102).

### 5.2 Carbohydrates

Although complex carbohydrates are the main source of energy for microbiota bacteria, few studies have examined the specific effect of these macronutrients on the production and phenotype of BEVs. This gap may be attributed to the complexity of carbohydrate structures and the diverse metabolic pathways involved in their utilization by microbiota. Understanding these effects could provide valuable insights into how dietary fibers and polysaccharides modulate BEV production, influencing gut health and systemic metabolic balance. Lago et al. evaluated the effects of β-mannan administration on BEVs produced by *Clostridiales, Bacilli*, and *Enterobacteriaceae*, and found that not only was BEV production by the microbiota stimulated, but also their protein composition was modified, although the consequences on host health remain to be elucidated (103). Another recent hypothesis is that the BEV content of polysaccharide-degrading enzymes such as glycosidase is increased by fiber-rich diets. This effect could be beneficial for the maintenance of intestinal metabolic balance by transferring these enzymes to bacteria lacking them through cross-feeding systems (43).

### 5.3 Lipids

The influence of HFD on the composition and release of BEVs has been demonstrated by Choi et al. (67). These researchers found that lipid administration in mice altered the number, size, and content of vesicles, specifically by decreasing their size and increasing their LPS content. As discussed in previous sections, increased LPS content in BEVs may be a trigger for metabolic disorders such as obesity and diabetes by increasing systemic endotoxemia and altering energy homeostasis and immune balance. But it is not only the HFD that has an effect on BEVs; as with proteins, but individual fatty acids can also alter the vesicles released. Bacteria have the ability to modulate their membrane composition under cellular stress conditions, and considering that BEVs originate from the plasma membrane, their release and composition also depend on these external stimuli. For example, Tafti et al. observed that *Bacteroides fragilis* increased BEV production upon the addition of saturated fatty acids, while *Bacteroides thetaiotomicron* had the same effect when unsaturated fatty acids were added (104). Therefore, the plasticity and differential response of individual bacteria to each type of fatty acid makes it difficult to assess the influence of fatty acids on BEVs and their implications for the host. It seems that the most affected component of BEVs upon fat ingestion is also their lipids. Lipidomic studies are particularly important because they can reveal how different types of fatty acids influence the lipid composition of BEVs, shedding light on their role in cellular communication and systemic effects. These studies could also help identify specific lipid biomarkers associated with metabolic disorders, providing a deeper understanding of the mechanisms by which dietary fats impact gut health and overall metabolism. Therefore, although this shift in research is very pioneering, the few existing studies have already demonstrated the ability of diet to influence BEVs, probably through much more rapid mechanisms than those involving complete modulation of microbial abundances and composition. In this context, precision nutrition emerges as a powerful tool to design personalised dietary interventions. While initially precision nutrition was based on the integration of phenotypic characteristics, medical history and environmental factors of the individual, increasing omics analyses have allowed the addition of other factors such as genetic variants, epigenetic marks and microbiota profiles, among others (105). Therefore, the analysis of the molecular content of BEVs released by the microbiota will be fundamental to understand the variation in the profile of these vesicles in response to specific macronutrients and dietary patterns, allowing nutritional interventions to be designed for each patient to prevent local and systemic pathologies and to promote gut microbial community homeostasis and overall health.

## 6 Gaps and future remarks

Although decades have passed since the discovery of BEVs, it is only now that they have awakened interest in research due to breakthroughs in omics technologies that have enabled detailed characterization of their cargo, including proteins, lipids, and nucleic acids. Recent findings have also linked BEVs to critical roles in immune modulation, inter-bacterial communication, and the progression of metabolic disorders, highlighting their significance in both health and disease. Advances in omics techniques have probably made it possible to analyze the content and composition of these vesicles and to study their plasticity in response to different environmental factors. However, we are still far from mastering them. There are still no universal and standardized isolation techniques that allow the isolation of BEVs with the highest possible efficiency and integrity for their subsequent analysis and applications. Furthermore, much remains to be understood about their mechanism of biogenesis and release and the factors that regulate these processes. Understanding the stimuli that trigger increases in vesicle release is fundamental to understanding the behavior of bacteria in the microbiota. However, this knowledge is hampered by the high diversity, not only among the bacteria that make up the microbiota themselves, but also the differences between inter-individual microbial communities. Nevertheless, this review has collected different studies that have already demonstrated the potential and impact of these vesicles as a therapeutic strategy. On the other hand, we should not forget that the analysis of the molecular cargo of these vesicles by bioinformatics analysis is crucial to better understand the impact on host health. The continued development of novel bioinformatics techniques will be crucial in the coming years to characterize BEVs released by different microbial phyla using multi-omics approaches. In addition, another essential focus of future research should be to discover the effect of food-derived vesicles on the composition and functionality of the microbial community. Despite all the current limitations, BEVs offer immense therapeutic potential for the attenuation of many diseases. However, further research is needed to determine the impact of dietary patterns on BEVs and their involvement in obesity. This knowledge will enable the design of nutritional strategies aimed at maintaining gut microbial balance and, consequently, systemic physiological homeostasis. These efforts could generate a new impetus for advancing precision nutrition and improving health outcomes.

## References

[1] Balakrishnan R Kang SI Lee JY Rho YK Kim BK Choi DK. Gut microbiota-immune system interactions in health and neurodegenerative diseases: insights into molecular mechanisms and therapeutic applications. Aging Dis. (2024). doi: 10.14336/AD.2024.1362 PMC12539548 39656490

[2] Yan J Yang L Ren Q Zhu C Du H Wang Z. Gut microbiota as a biomarker and modulator of anti-tumor immunotherapy outcomes. Front Immunol. (2024) 15:1471273. doi: 10.3389/fimmu.2024.1471273 39669573 PMC11634861

[3] Patloka O Komprda T Franke G. Review of the relationships between human gut microbiome, diet, and obesity. Nutrients. (2024) 16:3996. doi: 10.3390/nu16233996 39683390 PMC11643520

[4] Wang Y Bai M Peng Q Li L Tian F Guo Y. Angiogenesis, a key point in the association of gut microbiota and its metabolites with disease. Eur J Med Res. (2024) 29:614. doi: 10.1186/s40001-024-00614-0 39710789 PMC11664877

[5] Li Z Wang Q Huang X Wu Y Shan D. Microbiome's role in musculoskeletal health through the gut-bone axis insights. Gut Microbes. (2024) 16:2410478. doi: 10.1080/19490976.2024.2410478 39387683 PMC11469435

[6] Olasunkanmi OI Aremu J Wong ML Licinio J Zheng P. Maternal gut-microbiota impacts the influence of intrauterine environmental stressors on the modulation of human cognitive development and behavior. J Psychiatr Res. (2024) 180:307–26. doi: 10.1016/j.jpsychires.2024.03.012 39488009

[7] Ciernikova S Sevcikova A Mego M. Targeting the gut and tumor microbiome in cancer treatment resistance. Am J Physiol Cell Physiol. (2024) 327:C1433–50. doi: 10.1152/ajpcell.2024.00327.2024 39437444

[8] Wang Z Kaplan RC Burk RD Qi Q. The oral microbiota, microbial metabolites, and immuno-inflammatory mechanisms in cardiovascular disease. Int J Mol Sci. (2024) 25:12337. doi: 10.3390/ijms252212337 39596404 PMC11594421

[9] Azari H George M Albracht-Schulte K. Gut microbiota-microRNA interactions and obesity pathophysiology: A systematic review of integrated studies. Int J Mol Sci. (2024) 25:12836. doi: 10.3390/ijms252312836 39684547 PMC11640985

[10] Fujimura KE Slusher NA Cabana MD Lynch SV. Role of the gut microbiota in defining human health. Expert Rev Anti Infect Ther. (2010) 8:435–54. doi: 10.1586/eri.10.21 PMC2881665 20377338

[11] Adak A Khan MR. An insight into gut microbiota and its functionalities. Cell Mol Life Sci. (2019) 76:473–93. doi: 10.1007/s00018-018-2943-4 PMC11105460 30317530

[12] Díaz-Garrido N Badia J Baldomà L. Microbiota-derived extracellular vesicles in interkingdom communication in the gut. J Extracell Vesicles. (2021) 10:e12161. doi: 10.1002/jev2.12161 34738337 PMC8568775

[13] Cuesta CM Guerri C Ureña J Pascual M. Role of microbiota-derived extracellular vesicles in gut-brain communication. Int J Mol Sci. (2021) 22:4235. doi: 10.3390/ijms22084235 33921831 PMC8073592

[14] Brown L Wolf JM Prados-Rosales R Casadevall A. Through the wall: extracellular vesicles in Gram-positive bacteria, mycobacteria and fungi. Nat Rev Microbiol. (2015) 13:620–30. doi: 10.1038/nrmicro3480 PMC4860279 26324094

[15] Volgers C Savelkoul PHM Stassen FRM. Gram-negative bacterial membrane vesicle release in response to the host-environment: different threats, same trick? Crit Rev Microbiol. (2018) 44:258–73. doi: 10.1080/1040841X.2017.1366907 28741415

[16] Deatherage BL Lara JC Bergsbaken T Barrett SLR Lara S Cookson BT. Biogenesis of bacterial membrane vesicles. Mol Microbiol. (2009) 72:1395–407. doi: 10.1111/j.1365-2958.2009.06731.x PMC2745257 19432795

[17] Bernadac A Gavioli M Lazzaroni JC Raina S Lloubès R. Escherichia coli tol-pal mutants form outer membrane vesicles. J Bacteriol. (1998) 180:4872–8. doi: 10.1128/jb.180.18.4872-4878.1998 PMC107512 9733690

[18] Rowley G Spector M Kormanec J Roberts M. Pushing the envelope: extracytoplasmic stress responses in bacterial pathogens. Nat Rev Microbiol. (2006) 4:383–94. doi: 10.1038/nrmicro1384 16715050

[19] MacDonald IA Kuehn MJ. Stress-induced outer membrane vesicle production by Pseudomonas aeruginosa. J Bacteriol. (2013) 195:2971–81. doi: 10.1128/JB.02267-12 PMC3697536 23625841

[20] Schertzer JW Whiteley M. A bilayer-couple model of bacterial outer membrane vesicle biogenesis. MBio. (2012) 3:e00003–12. doi: 10.1128/mBio.00003-12 PMC3312216 22415005

[21] Gill S Catchpole R Forterre P. Extracellular membrane vesicles in the three domains of life and beyond. FEMS Microbiol Rev. (2019) 43:273–303. doi: 10.1093/femsre/fuy042 30476045 PMC6524685

[22] Sultan S Mottawea W Yeo J Hammami R. Gut microbiota extracellular vesicles as signaling molecules mediating host-microbiota communications. Int J Mol Sci. (2021) 22:12489. doi: 10.3390/ijms222312489 34884969 PMC8658398

[23] Toyofuku M Schild S Kaparakis-Liaskos M Eberl L. Composition and functions of bacterial membrane vesicles. Nat Rev Microbiol. (2023) 21:415–30. doi: 10.1038/s41579-023-00785-7 36932221

[24] van Niel G D’Angelo G Raposo G. Shedding light on the cell biology of extracellular vesicles. Nat Rev Mol Cell Biol. (2018) 19:213–8. doi: 10.1038/nrm.2017.125 29339798

[25] Jeppesen DK Zhang Q Franklin JL Coffey RJ. Extracellular vesicles and nanoparticles: emerging complexities. Trends Cell Biol. (2023) 33:667–81. doi: 10.1016/j.tcb.2023.05.006 PMC10363204 36737375

[26] Jang KS Sweredoski MJ Graham RLJ Hess S Clemons WM. Comprehensive proteomic profiling of outer membrane vesicles from Campylobacter jejuni. J Proteomics. (2014) 98:90–8. doi: 10.1016/j.jprot.2013.12.010 PMC4534003 24382552

[27] Nevermann J Silva A Otero C Oyarzún DP Barrera B Gil F. Identification of genes involved in biogenesis of outer membrane vesicles (OMVs) in Salmonella enterica Serovar Typhi. Front Microbiol. (2019) 10:104. doi: 10.3389/fmicb.2019.00104 30778340 PMC6369716

[28] Dean SN Leary DH Sullivan CJ Oh E Walper SA. Isolation and characterization of Lactobacillus-derived membrane vesicles. Sci Rep. (2019) 9:877. doi: 10.1038/s41598-018-37398-9 30696852 PMC6351534

[29] Dean SN Rimmer MA Turner KB Phillips DA Caruana JC Hervey WJ. Lactobacillus acidophilus membrane vesicles as a vehicle of bacteriocin delivery. Front Microbiol. (2020) 11:593761. doi: 10.3389/fmicb.2020.593761 PMC7203471 32425905

[30] Barák I Muchová K. The role of lipid domains in bacterial cell processes. Int J Mol Sci. (2013) 14:4050–65. doi: 10.3390/ijms14024050 PMC3588084 23429192

[31] Roier S Zingl FG Cakar F Durakovic S Kohl P Eichmann TO. A novel mechanism for the biogenesis of outer membrane vesicles in Gram-negative bacteria. Nat Commun. (2016) 7:10515. doi: 10.1038/ncomms10515 26806181 PMC4737802

[32] Bitto NJ Chapman R Pidot S Costin A Lo C Choi J. Bacterial membrane vesicles transport their DNA cargo into host cells. Sci Rep. (2017) 7:7072. doi: 10.1038/s41598-017-07288-4 28765539 PMC5539193

[33] Domingues S Nielsen KM. Membrane vesicles and horizontal gene transfer in prokaryotes. Curr Opin Microbiol. (2017) 38:16–21. doi: 10.1016/j.mib.2017.03.012 28441577

[34] Koeppen K Hampton TH Jarek M Scharfe M Gerber SA Mielcarz DW. A novel mechanism of host-pathogen interaction through sRNA in bacterial outer membrane vesicles. PloS Pathog. (2016) 12:e1005672. doi: 10.1371/journal.ppat.1005672 27295279 PMC4905634

[35] Celluzzi A Masotti A. How our other genome controls our epi-genome. Trends Microbiol. (2016) 24:777–87. doi: 10.1016/j.tim.2016.05.005 27289569

[36] Orench-Rivera N Kuehn MJ. Environmentally controlled bacterial vesicle-mediated export. Cell Microbiol. (2016) 18:1525–36. doi: 10.1111/cmi.12676 PMC5308445 27673272

[37] Taitz JJ Tan JK Potier-Villette C Ni D King NJC Nanan R. Diet, commensal microbiota-derived extracellular vesicles, and host immunity. Eur J Immunol. (2023) 53:e2360101. doi: 10.1002/eji.2022360101 37137164

[38] Yáñez-Mó M Siljander PRM Andreu Z Zavec AB Borràs FE Buzas EI. Biological properties of extracellular vesicles and their physiological functions. J Extracell Vesicles. (2015) 4:27066. doi: 10.3402/jev.v4.27066 25979354 PMC4433489

[39] Liang X Dai N Sheng K Lu H Wang J Chen L. Gut bacterial extracellular vesicles: important players in regulating intestinal microenvironment. Gut Microbes. (2022) 14:1–30. doi: 10.1080/19490976.2022.1987763 PMC9578468 36242585

[40] Stentz R Horn N Cross K Salt L Brearley C Livermore DM. Cephalosporinases associated with outer membrane vesicles released by Bacteroides spp. protect gut pathogens and commensals against β-lactam antibiotics. J Antimicrob Chemother. (2015) 70:701–9. doi: 10.1093/jac/dku468 PMC4319488 25433011

[41] Mukherjee S Bassler BL. Bacterial quorum sensing in complex and dynamically changing environments. Nat Rev Microbiol. (2019) 17:371–82. doi: 10.1038/s41579-019-0186-5 PMC6615036 30944413

[42] Cooke AC Nello AV Ernst RK Schertzer JW. Analysis of Pseudomonas aeruginosa biofilm membrane vesicles supports multiple mechanisms of biogenesis. PloS One. (2019) 14:e0212275. doi: 10.1371/journal.pone.0212275 30763382 PMC6375607

[43] Valguarnera E Scott NE Azimzadeh P Feldman MF. Surface exposure and packing of lipoproteins into outer membrane vesicles are coupled processes in Bacteroides. mSphere. (2018) 3:e00559–18. doi: 10.1128/mSphere.00559-18 PMC6222051 30404931

[44] Mashburn LM Whiteley M. Membrane vesicles traffic signals and facilitate group activities in a prokaryote. Nature. (2005) 437:422–5. doi: 10.1038/nature03925 16163359

[45] Ciofu O Beveridge TJ Kadurugamuwa J Walther-Rasmussen J Høiby N. Chromosomal β-lactamase is packaged into membrane vesicles and secreted from Pseudomonas aeruginosa. J Antimicrob Chemother. (2000) 45:9–13. doi: 10.1093/jac/45.1.9 10629007

[46] Schulz E Goes A Garcia R Panter F Koch M Müller R. Biocompatible bacteria-derived vesicles show inherent antimicrobial activity. J Control Release. (2018) 290:46–55. doi: 10.1016/j.jconrel.2018.10.029 30292423

[47] Cooke AC Florez C Dunshee EB Lieber AD Terry ML Light CJ. Pseudomonas quinolone signal-induced outer membrane vesicles enhance biofilm dispersion in Pseudomonas aeruginosa. mSphere. (2020) 5:e01109–20. doi: 10.1128/mSphere.01109-20 PMC7690959 33239369

[48] Li X Sun L Li C Yang X Wang X Hu X. The attenuated protective effect of outer membrane vesicles produced by an mcr-1 positive strain on colistin sensitive Escherichia coli. Front Cell Infect Microbiol. (2021) 11:664499. doi: 10.3389/fcimb.2021.664499 PMC8355893 34395312

[49] Kulkarni HM Nagaraj R Jagannadham MV. Protective role of E. coli outer membrane vesicles against antibiotics. Microbiol Res. (2015) 181:1–7. doi: 10.1016/j.micres.2015.08.004 26640046

[50] Yaron S Kolling GL Simon L Matthews KR. Vesicle-mediated transfer of virulence genes from Escherichia coli O157:H7 to other enteric bacteria. Appl Environ Microbiol. (2000) 66:4414–20. doi: 10.1128/AEM.66.10.4414-4420.2000 PMC92318 11010892

[51] Elhenawy W Debelyy MO Feldman MF. Preferential packing of acidic glycosidases and proteases into Bacteroides outer membrane vesicles. MBio. (2014) 5:e00909–14. doi: 10.1128/mBio.00909-14 PMC3952158 24618254

[52] Rompikuntal PK Vdovikova S Duperthuy M Johnson TL Åhlund M Lundmark R. Outer membrane vesicle-mediated export of processed PrtV protease from Vibrio cholerae. PloS One. (2015) 10:e0134098. doi: 10.1371/journal.pone.0134098 26222047 PMC4519245

[53] Bitar A Aung KM Wai SN Hammarström ML. Vibrio cholerae-derived outer membrane vesicles modulate the inflammatory response of human intestinal epithelial cells by inducing microRNA-146a. Sci Rep. (2019) 9:7210. doi: 10.1038/s41598-019-43677-y 31076615 PMC6510749

[54] Nishiyama K Takaki T Sugiyama M Fukuda I Aiso M Mukai T. Extracellular vesicles produced by Bifidobacterium longum. Infect Immun. (2020) 86:e00003–20. doi: 10.1128/IAI.00003-20 PMC7499026 32737132

[55] Lekmeechai S Su YC Brant M Alvarado-Kristensson M Vallström A Obi I. Helicobacter pylori outer membrane vesicles protect the pathogen from reactive oxygen species of the respiratory burst. Front Microbiol. (2018) 9:1837. doi: 10.3389/fmicb.2018.01837 30245670 PMC6137165

[56] Yonezawa H Osaki T Kurata S Fukuda M Kawakami H Ochiai K. Outer membrane vesicles of Helicobacter pylori TK1402 are involved in biofilm formation. BMC Microbiol. (2009) 9:197. doi: 10.1186/1471-2180-9-197 19751530 PMC2749055

[57] Gilmore WJ Bitto NJ Kaparakis-Liaskos M. Pathogenesis mediated by bacterial membrane vesicles. Subcell Biochem. (2021) 97:101–50. doi: 10.1007/978-3-030-67171-6_4 33779916

[58] Schetters STT Jong WSP Horrevorts SK Kruijssen LJW Engels S Stolk D. Outer membrane vesicles engineered to express membrane-bound antigen program dendritic cells for cross-presentation to CD8+ T cells. Acta Biomater. (2019) 91:248–57. doi: 10.1016/j.actbio.2019.03.050 31003032

[59] Shen Y Torchia MLG Lawson GW Karp CL Ashwell JD Mazmanian SK. Outer membrane vesicles of a human commensal mediate immune regulation and disease protection. Cell Host Microbe. (2012) 12:509–20. doi: 10.1016/j.chom.2012.08.004 PMC3895402 22999859

[60] Kang CS Ban M Choi EJ Moon HG Jeon JS Kim DK. Extracellular vesicles derived from gut microbiota, especially Akkermansia muciniphila, protect the progression of dextran sulfate sodium-induced colitis. PloS One. (2013) 8:e76520. doi: 10.1371/journal.pone.0076520 24204633 PMC3811976

[61] Chelakkot C Choi Y Kim DK Park HT Ghim J Kwon Y. Akkermansia muciniphila-derived extracellular vesicles influence gut permeability through the regulation of tight junctions. Exp Mol Med. (2018) 50:e450. doi: 10.1038/emm.2017.282 29472701 PMC5903829

[62] Yaghoubfar R Behrouzi A Zare Banadkoki E Ashrafian F Lari A Vaziri F. Effect of Akkermansia muciniphila, Faecalibacterium prausnitzii, and their extracellular vesicles on the serotonin system in intestinal epithelial cells. Probiotics Antimicrob Proteins. (2021) 13:1546–56. doi: 10.1007/s12602-021-09834-7 33852147

[63] Ashrafian F Shahriary A Behrouzi A Moradi HR Keshavarz Azizi Raftar S Lari A. Akkermansia muciniphila-derived extracellular vesicles as a mucosal delivery vector for amelioration of obesity in mice. Front Microbiol. (2019) 10:2155. doi: 10.3389/fmicb.2019.02155 31632356 PMC6779730

[64] Alvarez CS Badia J Bosch M Giménez R Baldomà L. Outer membrane vesicles and soluble factors released by probiotic Escherichia coli Nissle 1917 and commensal ECOR63 enhance barrier function by regulating expression of tight junction proteins in intestinal epithelial cells. Front Microbiol. (2016) 7:1981. doi: 10.3389/fmicb.2016.01981 28018313 PMC5156689

[65] Cañas MA Fábrega MJ Giménez R Badia J Baldomà L. Outer membrane vesicles from probiotic and commensal Escherichia coli activate NOD1-mediated immune responses in intestinal epithelial cells. Front Microbiol. (2018) 9:498. doi: 10.3389/fmicb.2018.00498 29616010 PMC5869251

[66] Fábrega MJ Rodríguez-Nogales A Garrido-Mesa J Algieri F Badía J Giménez R. Intestinal anti-inflammatory effects of outer membrane vesicles from Escherichia coli Nissle 1917 in DSS-experimental colitis in mice. Front Microbiol. (2017) 8:1274. doi: 10.3389/fmicb.2017.01274 28744268 PMC5504144

[67] Choi Y Kwon Y Kim DK Jeon J Jang SC Wang T. Gut microbe-derived extracellular vesicles induce insulin resistance, thereby impairing glucose metabolism in skeletal muscle. Sci Rep. (2015) 5:15878. doi: 10.1038/srep15878 26510393 PMC4625370

[68] Deo P Chow SH Han ML Speir M Huang C Schittenhelm RB. Mitochondrial dysfunction caused by outer membrane vesicles from Gram-negative bacteria activates intrinsic apoptosis and inflammation. Nat Microbiol. (2020) 5:1418–27. doi: 10.1038/s41564-020-00807-2 32807891

[69] Alpdundar Bulut E Bayyurt Kocabas B Yazar V Aykut G Guler U Salih B. Human gut commensal membrane vesicles modulate inflammation by generating M2-like macrophages and myeloid-derived suppressor cells. J Immunol. (2020) 205:2707–18. doi: 10.4049/jimmunol.2000606 33028617

[70] Hickey CA Kuhn KA Donermeyer DL Porter NT Jin C Cameron EA. Colitogenic Bacteroides thetaiotaomicron antigens access host immune cells in a sulfatase-dependent manner via outer membrane vesicles. Cell Host Microbe. (2015) 17:672–80. doi: 10.1016/j.chom.2015.04.002 PMC4432250 25974305

[71] Bryant WA Stentz R Le Gall G Sternberg MJE Carding SR Wilhelm T. In silico analysis of the small molecule content of outer membrane vesicles produced by Bacteroides thetaiotaomicron indicates an extensive metabolic link between microbe and host. Front Microbiol. (2017) 8:2440. doi: 10.3389/fmicb.2017.02440 29276507 PMC5727896

[72] Jones E Stentz R Telatin A Savva GM Booth C Baker D. The origin of plasma-derived bacterial extracellular vesicles in healthy individuals and patients with inflammatory bowel disease: a pilot study. Genes (Basel). (2021) 12:1578. doi: 10.3390/genes12101578 34681030 PMC8535827

[73] Wang X Ni J You Y Feng G Zhang S Bao W. SNX10-mediated LPS detection provokes intestinal barrier dysfunction through a caspase-5-dependent signaling cascade. EMBO J. (2021) 40:e108080. doi: 10.15252/embj.2021108080 34747049 PMC8672282

[74] Zubair M Abouelnazar FA Dawood AS Pan J Zheng X Chen T. Microscopic messengers: microbiota-derived bacterial extracellular vesicles in inflammatory bowel disease. Front Microbiol. (2024) 15:1481496. doi: 10.3389/fmicb.2024.1481496 39606115 PMC11600980

[75] Mohamed SM Shalaby MA El-Shiekh RA El-Banna HA Emam SR Bakr AF. Metabolic syndrome: risk factors, diagnosis, pathogenesis, and management with natural approaches. Food Chem Adv. (2023) 3:100246. doi: 10.1016/j.focha.2023.100246

[76] Hu X Yu C He Y Zhu S Wang S Xu Z. Integrative metagenomic analysis reveals distinct gut microbial signatures related to obesity. BMC Microbiol. (2024) 24:18. doi: 10.1186/s12866-024-02924-2 38580930 PMC10996249

[77] Sun L Ma L Ma Y Zhang F Zhao C Nie Y. Insights into the role of gut microbiota in obesity: pathogenesis, mechanisms, and therapeutic perspectives. Protein Cell. (2018) 9:397–403. doi: 10.1007/s13238-018-0549-2 29725936 PMC5960470

[78] Beam A Clinger E Hao L. Effect of diet and dietary components on the composition of the gut microbiota. Nutrients. (2021) 13:2795. doi: 10.3390/nu13082795 34444955 PMC8398149

[79] Jia L Vianna CR Fukuda M Berglund ED Liu C Tao C. Hepatocyte toll-like receptor 4 regulates obesity-induced inflammation and insulin resistance. Nat Commun. (2014) 5:3878. doi: 10.1038/ncomms4878 24815961 PMC4080408

[80] Ellis TN Leiman SA Kuehn MJ. Naturally produced outer membrane vesicles from Pseudomonas aeruginosa elicit a potent innate immune response via combined sensing of both lipopolysaccharide and protein components. Infect Immun. (2010) 78:3822–31. doi: 10.1128/IAI.00433-10 PMC2937433 20605984

[81] Kaisanlahti A Salmi S Kumpula S Amatya SB Turunen J Tejesvi M. Bacterial extracellular vesicles – brain invaders? A systematic review. Front Mol Neurosci. (2023) 16:1210234. doi: 10.3389/fnmol.2023.1210234 PMC10537964 37781094

[82] Yaghoubfar R Behrouzi A Ashrafian F Shahryari A Moradi HR Choopani S. Modulation of serotonin signaling/metabolism by Akkermansia muciniphila and its extracellular vesicles through the gut-brain axis in mice. Sci Rep. (2020) 10:10047. doi: 10.1038/s41598-020-66442-3 33335202 PMC7747642

[83] Emery DC Shoemark DK Batstone TE Waterfall CM Coghill JA Cerajewska TL. 16S rRNA next generation sequencing analysis shows bacteria in Alzheimer’s post-mortem brain. Front Aging Neurosci. (2017) 9:195. doi: 10.3389/fnagi.2017.00195 28676754 PMC5476743

[84] Al-Nedawi K Mian MF Hossain N Karimi K Mao YK Forsythe P. Gut commensal microvesicles reproduce parent bacterial signals to host immune and enteric nervous systems. FASEB J. (2015) 29:684–95. doi: 10.1096/fj.14-259598 25392266

[85] Palacios E Lobos-González L Guerrero S Kogan MJ Shao B Heinecke JW. Helicobacter pylori outer membrane vesicles induce astrocyte reactivity through nuclear factor-κappa B activation and cause neuronal damage *in vivo* in a murine model. J Neuroinflammation. (2023) 20:154. doi: 10.1186/s12974-023-02701-1 36895046 PMC9996972

[86] Lee KE Kim JK Han SK Lee DY Lee HJ Yim SV. The extracellular vesicle of gut microbial Paenalcaligenes hominis is a risk factor for vagus nerve-mediated cognitive impairment. Microbiome. (2020) 8:107. doi: 10.1186/s40168-020-00876-y 32669127 PMC7364628

[87] Pan J Wang Z Huang X Xue J Zhang S Guo X. Bacteria-derived outer-membrane vesicles hitchhike neutrophils to enhance ischemic stroke therapy. Adv Mater. (2023) 35:e2302296. doi: 10.1002/adma.202302296 37358255

[88] Zakharzhevskaya NB Vanyushkina AA Altukhov IA Shavarda AL Butenko IO Rakitina DV. Outer membrane vesicles secreted by pathogenic and nonpathogenic Bacteroides fragilis represent different metabolic activities. Sci Rep. (2017) 7:5008. doi: 10.1038/s41598-017-05264-7 28694488 PMC5503946

[89] Paolone G. From the gut to the brain and back: therapeutic approaches for the treatment of network dysfunction in Parkinson’s disease. Front Neurol. (2020) 11:557928. doi: 10.3389/fneur.2020.557928 33117258 PMC7575743

[90] Han EC Choi SY Lee Y Park JW Hong SH Lee HJ. Extracellular RNAs in periodontopathogenic outer membrane vesicles promote TNF-α production in human macrophages and cross the blood-brain barrier in mice. FASEB J. (2019) 33:13412–22. doi: 10.1096/fj.201901663R PMC6894046 31545910

[91] Zhan X. Author response: Gram-negative bacterial molecules associate with Alzheimer disease pathology. Neurology. (2017) 88:2338. doi: 10.1212/WNL.0000000000003997 28607142

[92] Choi J Kim YK Han PL. Extracellular vesicles derived from Lactobacillus plantarum increase BDNF expression in cultured hippocampal neurons and produce antidepressant-like effects in mice. Exp Neurobiol. (2019) 28:158–71. doi: 10.5607/en.2019.28.2.158 PMC6526105 31138987

[93] Filiano AJ Xu Y Tustison NJ Marsh RL Baker W Smirnov I. Unexpected role of interferon-γ in regulating neuronal connectivity and social behaviour. Nature. (2016) 535:425–9. doi: 10.1038/nature18626 PMC4961620 27409813

[94] Pinart M Schlicht K Laudes M Bouwman J Forslund SK Pischon T. Gut microbiome composition in obese and non-obese persons: A systematic review and meta-analysis. Nutrients. (2022) 14:1–41. doi: 10.3390/nu14122556 PMC8746372 35010887

[95] Rosés C Cuevas-Sierra A Quintana S Riezu-Boj JI Alfredo Martínez J Milagro FI. Gut microbiota bacterial species associated with Mediterranean diet-related food groups in a northern Spanish population. Nutrients. (2021) 13:1–17. doi: 10.3390/nu13020636 PMC7920039 33669303

[96] Rivero-Pino F Marquez-Paradas E Montserrat-de-la-Paz S. Food-derived vesicles as immunomodulatory drivers: current knowledge, gaps, and perspectives. Food Chem. (2024) 457:140168. doi: 10.1016/j.foodchem.2023.140168 38908244

[97] Tan J Ni D Taitz J Pinget GV Read M Senior A. Dietary protein increases T-cell-independent sIgA production through changes in gut microbiota-derived extracellular vesicles. Nat Commun. (2022) 13:7318. doi: 10.1038/s41467-022-34985-y 35896537 PMC9329401

[98] Luck H Khan S Kim JH Copeland JK Revelo XS Tsai S. Gut-associated IgA+ immune cells regulate obesity-related insulin resistance. Nat Commun. (2019) 10:3657. doi: 10.1038/s41467-019-11448-7 31409776 PMC6692361

[99] Hirayama S Nakao R. Glycine significantly enhances bacterial membrane vesicle production: a powerful approach for isolation of LPS-reduced membrane vesicles of probiotic Escherichia coli. Microb Biotechnol. (2020) 13:1162–78. doi: 10.1111/1751-7915.13536 PMC7264892 32348028

[100] van de Waterbeemd B Zomer G van den IJssel J van Keulen L Eppink MH van der Ley P. Cysteine depletion causes oxidative stress and triggers outer membrane vesicle release by Neisseria meningitidis: implications for vaccine development. PloS One. (2013) 8:e54314. doi: 10.1371/journal.pone.0054314 23372704 PMC3553081

[101] Sampath V McCaig WD Thanassi DG. Amino acid deprivation and central carbon metabolism regulate the production of outer membrane vesicles and tubes by Francisella. Mol Microbiol. (2018) 107:523–41. doi: 10.1111/mmi.13898 29240272

[102] Cava F Lam H De Pedro MA Waldor MK. Emerging knowledge of regulatory roles of d-amino acids in bacteria. Cell Mol Life Sci. (2011) 68:817–33. doi: 10.1007/s00018-010-0562-1 PMC3037491 21161322

[103] Lagos L La Rosa SL Arntzen M Ånestad R Terrapon N Gaby JC. Isolation and characterization of extracellular vesicles secreted *in vitro* by porcine microbiota. Microorganisms. (2020) 8:983. doi: 10.3390/microorganisms8070983 32630095 PMC7409281

[104] Tafti ZSM Moshiri A Marvasti FE Tarashi S Khalili SFS Motahhary A. The effect of saturated and unsaturated fatty acids on the production of outer membrane vesicles from Bacteroides fragilis and Bacteroides thetaiotaomicron. Gastroenterol Hepatol Bed Bench. (2019) 12:155–62. PMC6536021 31191841

[105] Ramos-Lopez O Assmann TS Astudillo Muñoz EY Baquerizo-Sedano L Barrón-Cabrera E Bernal CA. Guidance and position of RINN22 regarding precision nutrition and nutriomics. Lifestyle Genomics. (2024). doi: 10.1159/000529485 PMC11844698 39617000

