# Corrigendum: Microbiota-derived extracellular vesicles: current knowledge, gaps, and challenges in precision nutrition

**DOI:** 10.3389/fimmu.2025.1599029

**Published:** 2025-05-15

**Authors:** Elvira Marquez-Paradas, Maria Torrecillas-Lopez, Luna Barrera-Chamorro, Jose L. del Rio-Vazquez, Teresa Gonzalez-de la Rosa, Sergio Montserrat-de la Paz

**Affiliations:** ^1^ Department of Medical Biochemistry, Molecular Biology, and Immunology, School of Medicine, University of Seville, Seville, Spain; ^2^ Instituto de Biomedicina de Sevilla, IBiS/Hospital Universitario Virgen del Rocio/CSIC/Universidad de Sevilla, Seville, Spain

**Keywords:** diet, immunity, immunonutrition, gut microbiota, gut-brain-axis

In the published article, there was an error in the **Funding** statement. "The author(s) declare financial support was received for the research, authorship, and/or publication of this article. This study was supported by the research Grant PID2022-138650OA-I00 (Ministry of Science and Innovation, Government of Spain, MCIN/AEI/10.13039/501100011033/FEDER, UE)." The correct **Funding** statement appears below.

"The author(s) declare financial support was received for the research, authorship, and/or publication of this article. This publication is part of the project PID2022-138650OA-I00, funded by MICIU/AEI/10.13039/501100011033 and by ERDF/EU."

The authors apologize for this error and state that this does not change the scientific conclusions of the article in any way. The original article has been updated.

